# Umbilical cord-derived CD362^+^ mesenchymal stromal cells for *E. coli* pneumonia: impact of dose regimen, passage, cryopreservation, and antibiotic therapy

**DOI:** 10.1186/s13287-020-01624-8

**Published:** 2020-03-13

**Authors:** Shahd Horie, Claire Masterson, Jack Brady, Paul Loftus, Emma Horan, Lisa O’Flynn, Steve Elliman, Frank Barry, Timothy O’Brien, John G. Laffey, Daniel O’Toole

**Affiliations:** 1 Anaesthesia, School of Medicine, National University of Ireland, Galway, Ireland; 2 Regenerative Medicine Institute, National University of Ireland, Galway, Ireland; 3 Orbsen Therapeutics Ltd., Galway, Ireland; 4 Medicine, School of Medicine, National University of Ireland, Galway, Ireland

**Keywords:** Mesenchymal stem cell, ARDS, Pneumonia, Cryopreservation, Passage, Antibiotics

## Abstract

**Background:**

Mesenchymal stromal cells (MSCs) demonstrate considerable promise for acute respiratory distress syndrome (ARDS) and sepsis. However, standard approaches to MSC isolation generate highly heterogeneous cell populations, while bone marrow (BM) constitutes a limited and difficult to access MSC source. Furthermore, a range of cell manufacturing considerations and clinical setting practicalities remain to be explored.

**Methods:**

Adult male rats were subject to *E. coli*-induced pneumonia and administered CD362^+^ umbilical cord (UC)-hMSCs using a variety of cell production and clinical relevance considerations. In series 1, animals were instilled with *E*. *coli* and randomized to receive heterogeneous BM or UC-hMSCs or CD362^+^ UC-hMSCs. Subsequent series examined the impact of concomitant antibiotic therapy, MSC therapeutic cryopreservation (cryopreserved vs fresh CD362^+^ UC-hMSCs), impact of cell passage on efficacy (passages 3 vs 5 vs 7 vs 10), and delay of administration of cell therapy (0 h vs 6 h post-injury vs 6 h + 12 h) following *E*. *coli* installation.

**Results:**

CD362^+^ UC-hMSCs were as effective as heterogonous MSCs in reducing *E*. *coli*-induced acute lung injury, improving oxygenation, decreasing bacterial load, reducing histologic injury, and ameliorating inflammatory marker levels. Cryopreserved CD362^+^ UC-hMSCs recapitulated this efficacy, attenuating *E*. *coli*-induced injury, but therapeutic relevance did not extend beyond passage 3 for all indices. CD362^+^ UC-hMSCs maintained efficacy in the presence of antibiotic therapy and rescued the animal from *E*. *coli* injury when delivered at 6 h + 12 h, following *E*. *coli* instillation.

**Conclusions:**

These translational studies demonstrated the efficacy of CD362^+^ UC-hMSCs, where they decreased the severity of *E*. *coli*-induced pneumonia, maintained efficacy following cryopreservation, were more effective at early passage, were effective in the presence of antibiotic therapy, and could continue to provide benefit at later time points following *E*. *coli* injury.

## Introduction

Human mesenchymal stem/stromal cells (hMSCs) are a promising therapeutic strategy for the treatment of acute respiratory distress syndrome (ARDS) [1], demonstrating beneficial effects in a number of pre-clinical models including pulmonary [2–4] and abdominal sepsis [5–7], ventilator-induced lung injury (VILI) [8], bleomycin-induced acute lung injury [9] and fibrosis [10]. We have previously shown that bone marrow (BM)-derived hMSCs enhance resolution of VILI [11] and attenuate *E. coli*-induced pneumonia severity [12]. Accordingly, BM-derived MSCs are in early phase clinical testing, with a phase 1 study demonstrating the safety of fresh allogeneic BM-derived hMSCs in patients with moderate to severe ARDS [13] and another study advancing to phase 2 [14] (http://clinicaltrials.gov/ct2/show/NCT02611609).

A key focus in current research efforts is on developing MSC therapeutics that can be scaled to facilitate larger cohort clinical testing. However, three issues have been identified in this regard. Firstly, current approaches to isolating MSCs rely largely on the separation of the “plastic adherent” component of the BM mononuclear cell (MNC) population, followed by elucidation of their cell surface marker profile and differentiation assays [15]. This generates a heterogeneous hMSC population that may increase batch-to-batch variability and may be insufficiently pure to meet emerging regulatory requirements for advanced therapeutic medicinal products (ATMPs). Our recent demonstration that BM-derived CD362^+^ hMSCs can ameliorate both ventilator [11] and *E. coli*-induced ARDS [16] provides proof of principle for efficacy of this defined MSC subpopulation. Secondly, there are limitations in regard to the BM as an MSC source, as harvesting requires an invasive procedure, and the proliferative capacity of MSCs from this source is relatively limited. Furthermore, the heterogeneity across donors may contribute to batch-to-batch variability. As such, human umbilical cord (hUC) tissue has a number of advantages over BM: hUC tissue is a plentiful and easily accessible biologic waste product that produces 10 times more early passage MSCs per cord than a bone marrow harvest, allowing generation of high numbers of early passage MSCs [17]. In addition, all hUC-MSCs are the same “age” thus reducing variability and potentially enhancing potency [18]. Thirdly, MSCs must retain viability and efficacy following cryopreservation, storage, and transport, to be feasible as a clinical therapy. Demonstration of efficacy when administered immediately following thawing is therefore a key translational step particularly because MSCs need to demonstrate efficacy in a variety of clinical settings.

We wished to address a number of issues that are key to clinical translation in these studies, characterizing the efficacy of UC-derived CD362^+^ hMSCs (trademark Orbcel-C®) in a preclinical model of *E. coli*-induced pneumonia in the rat. These cells have recently been published as having broadly similar immunomodulatory properties as the more traditionally employed plastic-adherence isolated BM-MSCs [19]. We tested the following hypotheses: (a) that CD362^+^ UC-hMSCs would be as effective as standard BM and UC-derived hMSCs in attenuating *E. coli* pneumonia, (b) there is a therapeutic limit to the passaging of the hMSC, (c) CD362^+^ UC-hMSCs would prove effective in antibiotic treated pneumonia, (d) cryopreservation of CD362^+^ UC-hMSCs would retain efficacy, and (e) that delayed therapy with CD362^+^ UC-hMSCs would be effective in *E. coli* pneumonia.

## Materials and methods

All work was approved by the Animal Care in Research Ethics Committee of the National University of Ireland, Galway, and conducted under license from the Health Products Regulatory Agency, Ireland. Specific pathogen-free adult male Sprague Dawley rats (Charles River Laboratories, Kent, UK) weighing between 300 and 450 g were used in all experiments.

### CD362 isolation from human umbilical cord tissue

The heterogeneous BM and UC-hMSC and UC-derived CD362^+^ hMSC cell populations were provided by Orbsen Therapeutics Ltd. (Galway, Ireland). CD362^+^ hUC-MSC were prepared by a protocol similar to the bone marrow MSC as previously described [16] and modified for umbilical cord tissue source (Supplemental S1). All hMSC populations were cultured at 37 °C, 95% humidity, 5% CO_2_, and hypoxic conditions of 2% O_2_, until 70–80% confluent, and then trypsinized and culture expanded to passage 3–4, whereupon they were trypsinized and resuspended in 300 μL of phosphate-buffered saline (PBS) for fresh delivery (all series). For cryofrozen delivery (series 3), a cryovial containing 1 × 10^7^ cryopreserved cells in 1 mL was quickly thawed with 9 mL of PBS. One milliliter of MSC suspension for each 100 g of animal was pelleted at 400×*g* for 5 min and resupended in 300 μL of PBS ready for administration. Trypan blue exclusion dye staining was performed intermittently immediately post-thaw, indicating viability of 94.3 ± 1.5%. Of note, CD362 levels rapidly diminish with passaging post-isolation, and at the time of administration, all cell populations had identical low expression (data not shown).

### In vivo experimental protocols

#### *E. coli*-induced lung injury

As previously described [20], adult male Sprague Dawley rats were anesthetized with isoflurane (Pfizer, Kent, UK), and 2 × 10^9^*E*. *coli* E5162 (serotype: O9 K30 H10) in a 300-μL PBS suspension was instilled into the trachea under direct vision, and the animals were allowed to recover [21].

### Experimental design

Thirty minutes following intra-tracheal instillation of *E*. *coli* bacteria, animals were randomized to receive 1 × 10^7^/kg hMSCs or PBS vehicle (300 μL) IV, and the degree of injury assessed at 48 h. *Series 1*: (i) vehicle; (ii) BM heterogeneous hMSCs; (iii) UC heterogeneous hMSCs; and (iv) CD362^+^ UC-hMSCs. *Series 2*: (i) vehicle; (ii) antibiotic ceftriaxone 100 mg/kg; (iii) CD362^+^ UC-hMSCs; and (iv) antibiotic ceftriaxone 100 mg/kg + CD362^+^ UC-hMSCs. *Series 3*: (i) vehicle; (ii) fresh CD362^+^ UC-hMSCs; (iii) frozen UC CD362^+^ hMSC. *Series 4*: (i) vehicle; (ii) CD362^+^ UC-hMSCs passage 3; (iii) CD362^+^ UC-hMSCs passage 5; (iv) CD362^+^ UC-hMSCs passage 7; and (v) CD362^+^ UC-hMSCs passage 10. *Series 5*: (i) vehicle; (ii) time (T) 0 h fresh CD362^+^ UC-MSCs; T6h fresh CD362^+^ UC-hMSCs; T6h + T12h fresh CD362^+^ UC-hMSCs.

### Assessment of lung injury and recovery

#### In vivo assessment

At 48 h post-*E*. *coli* pneumonia induction, animals were anesthetized with intraperitoneal ketamine 80 mg kg^−1^ (Ketalar™; Pfizer, Cork, Ireland) and xylazine 8 mg kg^−1^ (Xylapan™; Vetoquinol S.A., Lure Cedex, France). After confirmation of depth of anesthesia by paw clamp, intravenous access was obtained via tail vein. Surgical tracheostomy was performed, and the trachea was intubated with the insertion of a tracheostomy tube. Following intra-arterial access, anesthesia was maintained with alfaxalone (Alfaxan™; Vetoquinol S.A.) and paralysis with cisatracurium besylate (Tracrium™; GlaxoSmithKline PLC., London, UK), and mechanical ventilation was commenced. Arterial blood pressure, airway pressure, lung static compliance, and arterial blood gas analyses were performed as previously described [22, 23].

#### Ex vivo analyses

Following exsanguination under anesthesia, bronchoalveolar lavage (BAL) was performed, and BAL fluid differential leukocyte counts and lung bacterial colony counts were performed. BAL concentrations of IL-1β, CINC-1, and IL-6 were determined using ELISA (R&D Systems, Abingdon, UK).

### Statistical analysis

Data was analyzed using Sigma Stat (SYSTAT® software, Richmond, CA, USA). The distribution of all data was tested for normality using Kolmogorov-Smirnov tests. Data were analyzed by one-way ANOVA, with post hoc testing using Dunnett’s test with the vehicle group as the single comparison group or with Student-Newman-Keuls between group comparisons as appropriate. Underlying model assumptions were deemed appropriate on the basis of suitable residual plots. A two-tailed *P* value of < 0.05 was considered significant.

## Results

All animals in each series survived the *E*. *coli* instillation and the recovery period. There were no significant differences between the groups at baseline in terms of pre-injury variables or the amount of instilled *E*. *coli* bacteria.

### Series 1—Efficacy of UC CD362^+^ hMSCs in *E. coli* lung injury

**Fig. 1:**
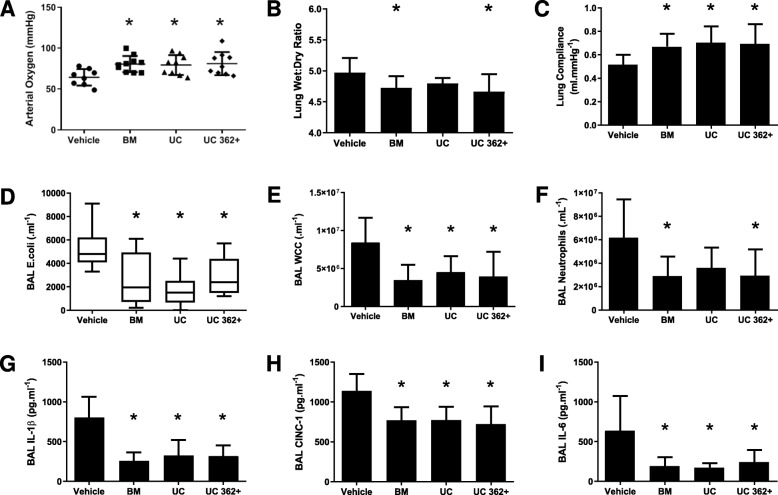
CD362^+^ UC-hMSCs decrease *E. coli*-induced lung injury. CD362^+^ UC-hMSCs ameliorated the decrement in arterial oxygenation to a similar degree as heterogeneous BM or UC-hMSC therapy (**a**). Heterogeneous BM and CD362^+^ UC-hMSC reduced the wet to dry ratio of lung tissue (**b**), while all hMSC types restored the decrement in static lung compliance (**c**), reduced lung *E*. *coli* bacterial load (**d**), and dampened total infiltrating cell count in BAL (**e**) compared to vehicle. Heterogeneous BM and UC CD362^+^, but not heterogeneous UC-hMSC significantly reduced BAL neutrophil counts (**f**). All 3 cell types attenuated the increase in BAL IL-1β (**g**), CINC-1 (**h**), and IL-6 (**i**) concentrations. Abbreviations: vehicle, treatment with vehicle alone; BM, bone marrow-derived heterogeneous hMSC; UC, umbilical cord-derived heterogeneous hMSC; UC 362+, umbilical cord-derived CD362^+^ hMSC; BAL, bronchoalveolar lavage; IL-1β, interleukin 1 beta; CINC-1, cytokine-induced neutrophil chemoattractant 1; IL-6, interleukin 6. Error bars represent standard deviation. *Significantly (*P* < 0.05) different from the vehicle control group

Forty animals were entered into the experimental protocol, with 10 randomized to each group. All hMSC therapy decreased the severity of *E*. *coli*-induced lung injury compared to vehicle controls. Heterogeneous BM-derived, heterogeneous UC-derived, and CD362^+^ UC-hMSC therapy attenuated the decrease in arterial oxygenation that was seen in the vehicle animal controls (Fig. 1a). hMSCs attenuated the increase in lung microvascular permeability with the wet to dry ratio significantly reduced in the BM and CD362^+^ UC-hMSC groups (Fig. 1b). Static lung compliance was increased in all hMSC treatment groups (Fig. 1c), and BAL bacterial load was likewise significantly reduced in all treatment groups (Fig. 1d). In cytological evaluation of BAL, all hMSC therapy groups exhibited a decrease in total infiltrating cell count (Fig. 1e), although only BM and CD362^+^ UC-hMSCs were capable of reducing neutrophil numbers (Fig. 1f). All cell types were capable of ameliorating inflammatory cytokine secretion to the BAL, with significant reduction in IL-1β (Fig. 1g), CINC-1 (Fig. 1h), and IL-6 (Fig. 1i) concentrations. Histological analysis of lung tissue revealed an increase in airspace in all hMSC treatment groups compared to vehicle, with a corresponding amelioration of tissue percentage (Supplemental Figure S2). Representative images are also provided (Supplemental Figure S2A–D) respective to each experimental group.

### Series 2—Effect of CD362^+^ UC-hMSCs in the presence of antibiotic therapy

**Fig. 2:**
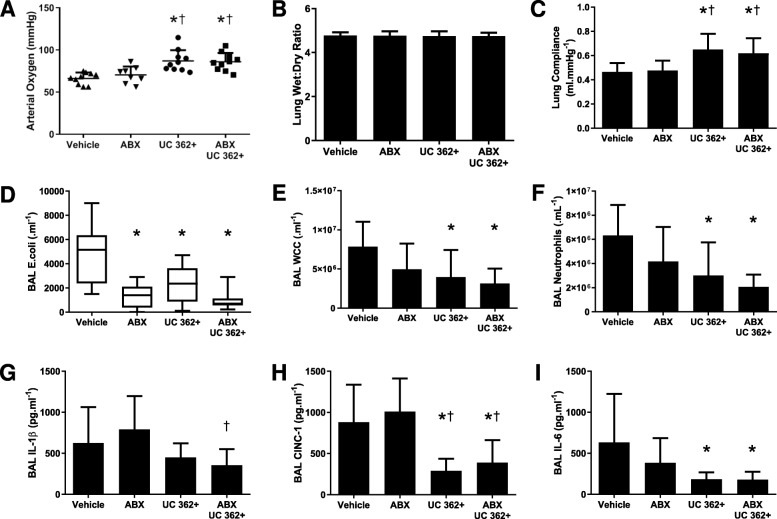
CD362^+^ UC-hMSCs decrease *E*. *coli*-induced lung injury in the presence of antibiotic therapy. CD362^+^ UC-hMSCs reduced the decrement in arterial oxygenation in the presence or absence of antibiotic therapy compared to vehicle, but antibiotic alone was ineffective (**a**). There was no effect of any treatment on lung wet to dry ratio (**b**). CD362^+^ UC-hMSCs, with or without antibiotics, reduced the decrease in lung static compliance (**c**), while all therapy groups reduced BAL *E*. *coli* bacterial load (**d**). CD362^+^ UC-hMSC therapy, with or without antibiotics, reduced both total leukocyte (**e**) and neutrophil (**f**) counts in the BAL, where antibiotic alone did not. No therapy regimen reduced IL-1β concentrations with respect to vehicle control, although CD362^+^ UC-hMSCs reduced levels compared to antibiotic therapy alone (**g**). CD362^+^ UC-hMSCs significantly reduced BAL CINC-1 (**h**), and IL-6 (**i**) concentrations in the presence or absence of antibiotic therapy where antibiotic therapy alone did not. Abbreviations: vehicle, treatment with vehicle alone; ABX, antibiotic therapy; UC 362+, umbilical cord-derived CD362^+^ hMSC. BAL, bronchoalveolar lavage; CINC-1, cytokine-induced neutrophil chemoattractant 1; IL-6, interleukin 6; IL-1β, interleukin 1 beta; vehicle, treatment with vehicle alone. Error bars represent standard deviation. *Significantly (*P* < 0.05) different from vehicle control group. ^†^Significantly (*P* < 0.05) different from the ABx therapy group

Forty animals were entered into the experimental protocol, with 10 allocated to each group. Over 48 h, CD362^+^ UC-hMSC therapy, with or without antibiotic, was more effective than antibiotic alone or vehicle, ameliorating *E. coli*-induced pneumonia injury. Both the CD362^+^ UC-hMSC and CD362^+^ UC-hMSC plus antibiotic groups significantly reduced the decrement in arterial oxygen pressure versus antibiotic and vehicle (Fig. 2a). No changes in wet to dry ratio were observed in this series (Fig. 2b), but both CD362^+^ UC-hMSC with or without antibiotic, ameliorated the reduction in static compliance (Fig. 2c). As might be expected, the BAL bacterial load of the CD362^+^ UC-hMSC and CD362^+^ UC-hMSC plus antibiotic groups were significantly reduced versus vehicle, but not when compared to the antibiotic control group (Fig. 2d). CD362^+^ UC-hMSC and CD362^+^ UC-hMSC plus antibiotic significantly reduced the total infiltrating cell count versus vehicle or antibiotic alone, but combination therapy was not superior to CD362^+^ UC-hMSC alone (Fig. 2e). Neutrophil count followed a similar pattern, with CD362^+^ UC-hMSC reducing numbers in the presence or absence of antibiotic to a comparable level (Fig. 2f). In BAL cytokine analysis, IL-1β levels were not significantly ameliorated compared to vehicle control by any regimen, although CD362^+^ UC-hMSC plus antibiotic was superior to antibiotic alone (Fig. 2g). CINC-1 and IL-6 levels were reduced with both CD362^+^ UC-hMSC and CD362^+^ UC-hMSC plus antibiotic therapy (Fig. 2h, i).

### Series 3—Effect of cryopreservation on CD362^+^ UC-hMSC therapy

**Fig. 3:**
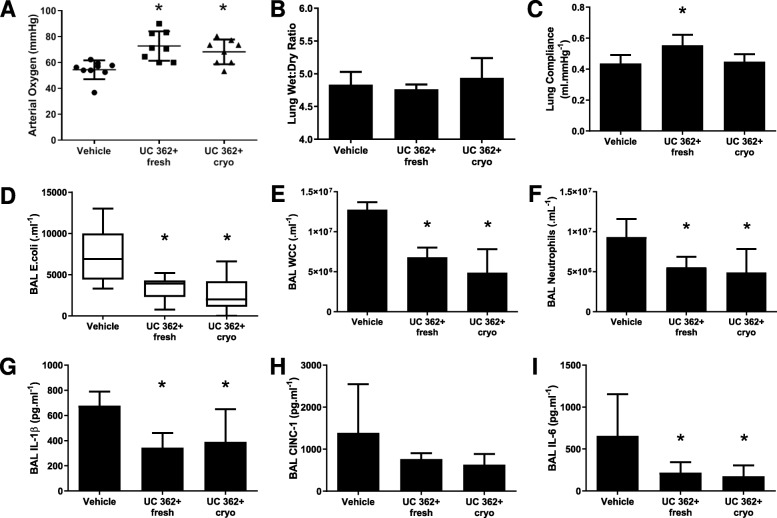
Freshly harvested or cryopreserved CD362^+^ UC-hMSCs have comparable therapeutic effects in *E*. *coli*-induced lung injury. Freshly harvested from culture or thawed from cryostorage and immediately administered, CD362^+^ UC-hMSCs ameliorated *E. coli* ARDS-induced decrement in arterial oxygenation (**a**). There were no significant changes in lung tissue wet to dry ratio (**b**). Freshly harvested CD362^+^ UC-hMSCs alone significantly reversed the decrease in lung static compliance (**c**). Both fresh and cryopreserved CD362^+^ UC-hMSCs reduced lung *E*. *coli* bacterial load (**d**), total BAL infiltrating leukocytes (**e**) BAL neutrophils (**f**), BAL IL-1β (**g**), and BAL IL-6 (**i**), and had a trending but not significant effect on CINC-1 (**h**) in this series. Abbreviations: vehicle, treatment with vehicle alone; UC 362^+^ fresh, umbilical cord-derived CD362^+^ hMSC freshly harvested from culture; UC 362^+^ cryo, umbilical cord-derived CD362^+^ hMSC thawed from cryostorage and immediately administered; BAL, bronchoalveolar lavage; IL-1β, interleukin 1 beta; CINC-1, cytokine-induced neutrophil chemoattractant 1; IL-6, interleukin 6. Error bars represent standard deviation. *Significantly (*P* < 0.05) different from vehicle control group

Twenty-four animals were entered into the experimental protocol, with 8 allocated to each group. The efficacy of the cryofrozen CD362^+^ UC-hMSCs was comparable to that seen with freshly harvested cells. hMSC therapy with freshly harvested or cryofrozen cells was equally effective in attenuating *E*. *coli*-induced pneumonia ARDS with the decrement in arterial oxygen pressure significantly reversed in both groups (Fig. 3a). No significant changes in the wet to dry ratio were observed in this series (Fig. 3b), while freshly harvested cells were more effective in reducing the decrement in static lung compliance (Fig. 3c). BAL bacteria CFU count (Fig. 3d), total BAL cell count (Fig. 3e), and BAL neutrophil count (Fig. 3f) were significantly decreased compared to PBS vehicle animals in both hMSC regimens. In BAL cytokine analysis, both CD362^+^ UC-hMSC fresh and frozen groups significantly reduced IL-1β (Fig. 3g) and IL-6 (Fig. 3i) concentrations compared to the PBS vehicle animals. CINC-1 trended towards decrease in both groups compared to controls (Fig. 3h) but did not reach significance.

### Series 4—Effect of passage on CD362^+^ UC-hMSCs

**Fig. 4:**
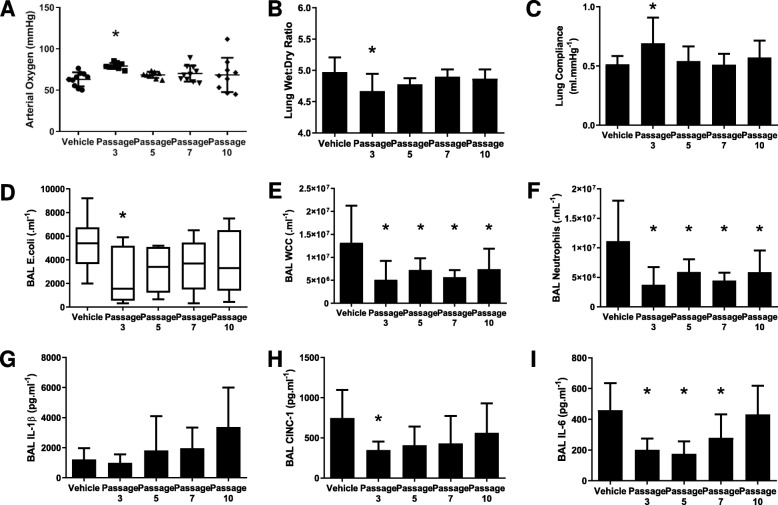
CD362^+^ UC-hMSCs lose function after passage 3 in *E. coli* pneumonia. Passage 3 CD362^+^ UC-hMSCs ameliorated the *E. coli*-induced decrement in arterial oxygenation (**a**), the increase in wet to dry ratio (**b**), the decrease in static lung compliance (**c**) and BAL bacterial load (**d**) compared to vehicle control. These effects were not observed at any later hMSC passage. All CD362^+^ UC-hMSC passages reduced the total infiltrating leukocyte (**e**) and neutrophil count (**f**) in BAL compared to the vehicle control. There was no effect of CD362^+^ UC-hMSCs on BAL IL-1β concentration (**h**) compared to the vehicle control. Passage 3 CD362^+^ UC-hMSCs ameliorated the *E. coli*-induced increase in BAL CINC-1 concentration (**i**) compared to the vehicle control, while passages 3 to 7 decreased IL-6 concentration (**j**). Abbreviations: vehicle, treatment with vehicle alone; passage, passage number of umbilical cord-derived CD362^+^ hMSC, BAL, bronchoalveolar lavage; IL-1β, interleukin 1 beta; CINC-1, cytokine-induced neutrophil chemoattractant 1; IL-6, interleukin 6. Error bars represent standard deviation. *Significantly (*P* < 0.05) different from the vehicle control group

Fifty animals were entered into the experimental protocol, with 10 allocated to each group. CD362^+^ UC-hMSC therapy with passage 3 cells ameliorated the *E. coli*-induced pneumonia, attenuating the decrement in arterial oxygen pressure, but the effect was lost at higher passage (Fig. 4a). In this instance, the increase in lung wet to dry ratio (Fig. 4b), the decrement in static lung compliance (Fig. 4c), and the total bacterial load in the lung (Fig. 4d) were significantly improved with passage 3 cells only, although bacterial clearance exhibited a strong trend at all hMSC passages. Interestingly, CD362^+^ UC-hMSC therapy decreased overall alveolar inflammatory cell infiltration (Fig. 4e) and substantially decreased neutrophil count (Fig. 4f) in the alveolar fluid at passage 3, 5, 7, and 10 treated animals, compared with the PBS vehicle animals. BAL cytokine concentrations were affected in a similar manner with administration of later passage CD362^+^ UC-hMSCs, with levels of the pro-inflammatory cytokine CINC-1 not ameliorated beyond hMSCs at passage 3 (Fig. 4h) and IL-6 levels not reduced beyond passage 7 (Fig. 4i). There were no significant changes in IL-1β concentration, though there was an observed trend towards increase with later passage (Fig. 4g).

### Series 5—Effect of dose timing and regimen on CD362^+^ UC-hMSC efficacy

**Fig. 5:**
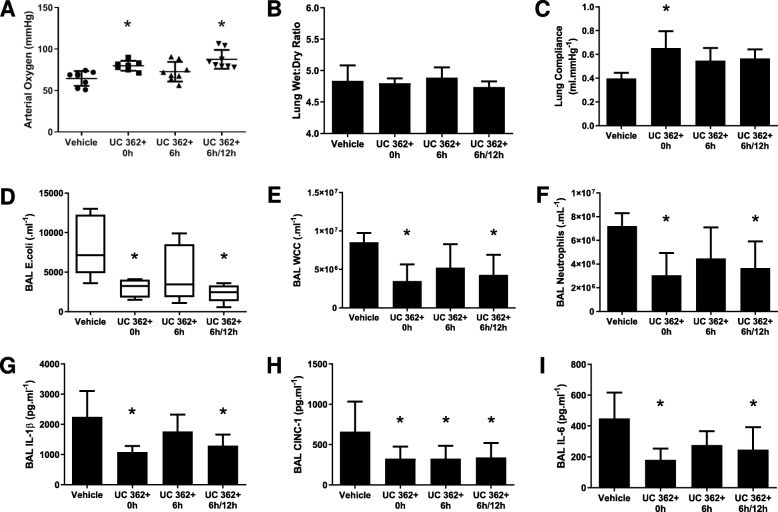
Repeat dosing with CD362^+^ UC-hMSCs extends the therapeutic window in *E. coli*-induced ARDS. CD362^+^ UC-hMSCs administered contemporaneously or at 6 h plus 12 h post-commencement of *E. coli* ARDS, ameliorated the decrement in arterial oxygenation (**a**), reduced lung *E. coli* bacterial load (**d**), total BAL infiltrating leukocytes (**e**) and neutrophils (**f**) compared to vehicle control or a single dose of CD362^+^ UC-hMSCs delivered 6 h post-induction. There were no significant changes in lung tissue wet to dry ratio (**b**), and the contemporaneous delivery alone significantly reversed the decrease in lung static compliance (**c**). CD362^+^ UC-hMSCs administered contemporaneously or at 6 h plus 12 h post-*E. coli* injury significantly reduced *E. coli* ARDS-induced levels of BAL inflammatory cytokines IL-1β (**h**), and BAL IL-6 (**j**), while each dosing regimen decreased BAL CINC-1 (**i**). Abbreviations: vehicle, treatment with vehicle alone; UC 362^+^, umbilical cord-derived CD362^+^ hMSC; 0 h, delivery at 0 h post-*E. coli* instillation alone; 6 h, delivered at 6 h post-*E. coli* instillation alone; 6 h + 12 h, delivered at 6 h and 12 h post-*E. coli* instillation; BAL, bronchoalveolar lavage; IL-1β, interleukin 1 beta; CINC-1, cytokine-induced neutrophil chemoattractant 1; IL-6, interleukin 6. Error bars represent standard deviation. *Significantly (*P* < 0.05) different from the vehicle control group

Thirty-two animals were entered into the experimental protocol, with 8 allocated to each of the groups. CD362^+^ UC-hMSC therapy administered at 6 h + 12 h was similar to that at delivered at 0 h, with both equally effective in attenuating the *E. coli*-induced ARDS, while one dose of hMSC therapy at 6 h time point was ineffective. Although again no changes were seen in wet to dry ratio (Fig. 5b), the decrements in arterial oxygen pressure (Fig. 5a) and static lung compliance (Fig. 5c) were significantly reversed. BAL bacterial load was significantly reduced in the 6 h + 12 h dual dosing group (Fig. 5d), while BAL total cell count (Fig. 5e) and BAL neutrophil count (Fig. 5f) were also significantly reduced compared to vehicle control animals. BAL inflammatory cytokines had a similar pattern, with IL-1β and IL-6 significantly reduced under either 0 h or 6 h + 12 h dosing regimen, but not with 6 h post-injury induction therapy alone (Fig. 5g, i). In contrast, CINC-1 was significantly reduced with all hMSC delivery groups (Fig. 5h).

## Discussion

In this study, we show that the severity of *E. coli*-induced pneumonia in a rodent model is effectively reduced following the administration of a specific CD362-positive subpopulation of umbilical cord-derived hMSCs. We demonstrate that these CD362^+^ UC-MSCs reduced lung damage and cleared the *E*. *coli* bacterial load from the lungs. The CD362^+^ UC-MSCs decreased both physiological indices and cytokine responses that are evident in *E. coli*-induced lung injury. Taken together, these data provide important insights regarding the therapeutic potential of a more homogeneous and easily sourced UC-derived CD362^+^ hMSC subpopulation for the treatment of *E*. *coli* pneumonia.

### Rationale for investigating specific hMSC subpopulations

This study continues our investigation of defined hMSC subpopulations, as previously shown using CD362^+^ MSCs from bone marrow [16] and now from UC sources. hMSCs isolated using specific cell surface antigens rather than conventional plastic adherence techniques have a number of potential advantages with regard to clinical translation. This includes the reduction of hMSC population heterogeneity, more thorough characterization in early isolation, and a better-defined MSC end product. These attributes pertain to the suggestion that heterogeneous hMSC populations may not be sufficiently defined to meet European Union (EU CAT/571134) and British Standard Institute (PAS-93) clinical grade standards according to the regulatory requirements for Advanced Therapeutic Medicinal Products for clinical use. Due to the heterogeneity of MSCs, the FDA’s MSC Consortium is focusing on methods of determining MSC activity and improving cell therapy product characterization. The use of a specific MSC marker would not only provide a more uniform MSC population to study, but would also feed into both the prediction of functionality and characterization of the product, fulfilling both of these objectives.

### CD362^+^ hMSC subpopulation

Minimum MSC characterization criteria according to the ISCT include the cell surface marker expression of CD105, 73, and 90 but not CD45, 34, 14, 11b, 79α, and 19, or HLA-DR [15]. Cell surface marker-based characterization is hindered because these and other currently described markers are not unique to MSCs [24] and the validity of this method has been called into question [25]. This has necessitated a drive to discover new cell surface markers to isolate potentially superior subpopulations of MSCs. As previously described, Orbsen Therapeutics Ltd. identified the CD362 protein (Syndecan-2) expressed on the surface of hMSCs as a novel isolation marker for BM-derived hMSCs [16]. Subsequently, the CD362 protein was found to be a novel isolation marker for MSCs derived from hUC tissue, which is a more accessible and advantageous source. As such, UC-derived CD362^+^ hMSCs were used in this study.

### CD362^+^ hMSCs improved *E*. *coli*-induced pneumonia

CD362^+^ UC-hMSCs attenuated the severity of *E. coli*-induced pneumonia acute lung injury, as demonstrated by a clear reduction in the presentation of impaired physiologic indices of lung dysfunction, such as an improvement in arterial blood oxygenation and restoration of lung compliance. Importantly, CD362^+^ UC-hMSCs appeared to be equally efficacious when compared to plastic adherence isolated (heterogeneous) BM- or UC-hMSCs, while remaining superior with regard to definition of medicinal product and availability of tissue source for isolation.

On presentation to the clinic, all patients diagnosed with sepsis are likely to be placed immediately on a regimen of broad spectrum antibiotics, as even recent advances in pathogen identification do not allow more tailored medicines until hours after the critical window of intervention has been passed. We have shown that CD362^+^ UC-MSC therapy is compatible with antibiotic use during pneumonia, in some cases superior to antibiotic use, and can potentially enhance the overall well-being of the patient beyond what is seen with antibiotics alone.

From a cell manufacturing perspective, we have now established that hMSC therapy may be subject to a limitation with regard to the dose size that can be generated from a single donor. It has previously been demonstrated that some therapy relevant properties of MSCs such as inhibition of T cell proliferation and CD73 expression do change over passage, but in these studies, we found that overall therapeutic efficacy in ARDS was lost beyond passage 3. Interestingly, some of the anti-inflammatory properties of the hMSCs may still be intact at these later passages, even though the demanding therapeutic needs of pneumonia-induced lung injury, requiring a multi-modal mechanism of action, are not addressed. This finding could have ramifications to specific potency assays for MSC batch release from cell therapy manufacturers and on practicalities of generating human scaled doses of these cell therapies.

Assessing the therapeutic window following pneumonia induction, we demonstrated that MSC effect was lost when cells were administered 6 h post-infection. However, the therapeutic window could be rescued by the administration of a further dose of MSCs at 12 h post-pneumonia induction. While there is evidence the MSC responds to the inflammatory environment and this can enhance therapeutic effect, our experiments would point to administration at the earliest possible opportunity as being superior. Injury is already evolving in our relatively rapid *E.coli* pneumonia model at 6 h, which makes us think the rescue of effect with the additional intervention is more due to the extra dose than the time of the dose, as, if anything, the earlier dose at 6 h would be the most prophylactic. However, clinical relevance is important, so it was vital we show efficacy of a dosing regimen in an already evolving pneumonia. In light of the recent somewhat disappointing START trial results [14], the option of repeat dosing may be an important consideration for future clinical trials, particularly where the patient is at a relatively advanced stage of ARDS [16].

Finally, we have shown that a cryopreserved stock of CD362^+^ UC-hMSCs is a viable strategy for use as an effective therapeutic in pneumonia. This is of critical importance in a rapid onset acute disease such as sepsis, where there is insufficient time to prepare a human-sized dose of hMSCs for fresh harvest from culture. Furthermore, cryopreserved hMSCs would further reduce inter-batch variability and ensure multiple doses of a homogenous end-therapy.

These data extend prior findings demonstrating the therapeutic potential of xenogeneic hMSC therapy in murine models of endotoxin [26] and bacterial lung injury [27] and in the ex vivo human lung [28]. We utilized a rodent *E. coli* model of lung injury in order to examine the efficacy of xenogeneic hMSC transplantation in immune competent animals and to determine the effect on lung injury severity, indices of inflammation, bacterial clearance, and histologic injury.

These studies provide insights into the therapeutic effects of UC-derived CD362^+^ hMSCs, yet additional studies are required particularly in terms of deciphering their mechanism(s) of therapeutic action. Furthermore, a homogenous cell population would indeed yield less variability but does not add to the assurance of their potency, and this also warrants further investigation. Other studies should also examine optimal routes and doses of administration to clarify the most opportune method for administering the lowest effective dose. Finally, caution as always must be taken when extrapolating from pre-clinical models to the clinical condition, particularly in xenogeneic cell therapy experiments.

## Conclusions

In these studies, we demonstrate that UC-derived CD362^+^ human MSCs transplanted xenogeneically into the immune competent rat reduces an *E. coli*-induced acute lung injury. We have also established that cryofrozen storage of the therapeutic is a viable option, as is administration in parallel with an antibiotic regimen. Taken together with our recent finding that BM-derived CD362^+^ hMSCs can ameliorate *E. coli*-induced injury, these data provide important insights regarding the therapeutic potential of a defined subset of hMSCs, specifically CD362^+^ for pneumonia.

## Supplementary information

**Additional file 1: Supplemental S1.** CD362 Isolation from Human Umbilical Cord Tissue. **Figure S1.** Isolation of CD362+ cells from human umbilical cords tissue by MACs. **Figure S2.** CD362^+^ UC-hMSCs reduce the severity of histologic injury following *E. coli*-induced lung injury.

## Data Availability

The datasets used and/or analyzed during the current study are available from the corresponding author on reasonable request.

## Abbreviations

ANOVA: Analysis of variance
ARDS: Acute respiratory distress syndrome
BAL: Bronchoalveolar lavage
BM: Bone marrow
CFU: Colony-forming units
CINC: Cytokine-induced neutrophil chemoattractant
ELISA: Enzyme-linked immunosorbent assay
HLA-DR: Human leukocyte antigen – DR isotype
IL: Interleukin
ISCT: International Society for Cellular Therapy
IV: Intravenous
MNC: Mononuclear cells
MSC: Mesenchymal stem/stromal cell
PBS: Phosphate-buffered saline
PLC: Public limited company
UC: Umbilical cord
UK: United Kingdom
VILI: Ventilator-induced lung injury

## References

[1] Gotts JE, Matthay MA (2011). Mesenchymal stem cells and acute lung injury. Crit Care Clin.

[2] Gupta N, Su X, Popov B, Lee J, Serikov V, Matthay M (2007). Intrapulmonary delivery of bone marrow-derived mesenchymal stem cells improves survival and attenuates endotoxin-induced acute lung injury in mice. J Immunol.

[3] Ionescu L, Byrne R, van Haaften T, Vadivel A, Alphonse R, Rey-Parra G (2012). Stem cell conditioned medium improves acute lung injury in mice: in vivo evidence for stem cell paracrine action. Am J Physiol Lung Cell Mol Physiol.

[4] Mao M, Wang S, Lv X, Wang Y, Xu J (2010). Intravenous delivery of bone marrow-derived endothelial progenitor cells improves survival and attenuates lipopolysaccharide-induced lung injury in rats. Shock..

[5] Mei S, Haitsma J, Dos Santos C, Deng Y, Lai P, Slutsky A (2010). Mesenchymal stem cells reduce inflammation while enhancing bacterial clearance and improving survival in sepsis. Am J Respir Crit Care Med.

[6] Krasnodembskaya A, Samarani G, Song Y, Zhuo H, Su X, Lee J (2012). Human mesenchymal stem cells reduce mortality and bacteremia in gram-negative sepsis in mice in part by enhancing the phagocytic activity of blood monocytes. Am J Physiol Lung Cell Mol Physiol.

[7] Nemeth K, Mayer B, Mezey E (2010). Modulation of bone marrow stromal cell functions in infectious diseases by toll-like receptor ligands. J Mole Med.

[8] Chimenti L, Luque T, Bonsignore MR, Ramirez J, Navajas D, Farre R (2012). Pre-treatment with mesenchymal stem cells reduces ventilator-induced lung injury. Eur Respir J.

[9] Aguilar S, Scotton CJ, McNulty K, Nye E, Stamp G, Laurent G (2009). Bone marrow stem cells expressing keratinocyte growth factor via an inducible lentivirus protects against bleomycin-induced pulmonary fibrosis. PLoS One.

[10] Ortiz LA, Gambelli F, McBride C, Gaupp D, Baddoo M, Kaminski N (2003). Mesenchymal stem cell engraftment in lung is enhanced in response to bleomycin exposure and ameliorates its fibrotic effects. Proc Natl Acad Sci U S A.

[11] Curley GF, Hayes M, Ansari B, Shaw G, Ryan A, Barry F (2012). Mesenchymal stem cells enhance recovery and repair following ventilator-induced lung injury in the rat. Thorax..

[12] Devaney J, Horie S, Masterson C, Elliman S, Barry F, O'Brien T (2015). Human mesenchymal stromal cells decrease the severity of acute lung injury induced by E. coli in the rat. Thorax..

[13] Wilson JG, Liu KD, Zhuo H, Caballero L, McMillan M, Fang X (2015). Mesenchymal stem (stromal) cells for treatment of ARDS: a phase 1 clinical trial. Lancet Respir Med.

[14] Matthay MA, Calfee CS, Zhuo H, Thompson BT, Wilson JG, Levitt JE (2019). Treatment with allogeneic mesenchymal stromal cells for moderate to severe acute respiratory distress syndrome (START study): a randomised phase 2a safety trial. Lancet Respir Med.

[15] Dominici M, Le Blanc K, Mueller I, Slaper-Cortenbach I, Marini F, Krause D (2006). Minimal criteria for defining multipotent mesenchymal stromal cells. The International Society for Cellular Therapy position statement. Cytotherapy..

[16] Masterson C, Devaney J, Horie S, O'Flynn L, Deedigan L, Elliman S (2018). Syndecan-2-positive, bone marrow-derived human mesenchymal stromal cells attenuate bacterial-induced acute lung injury and enhance resolution of ventilator-induced lung injury in rats. Anesthesiology..

[17] Hua Jie, Gong Jian, Meng Hongbo, Xu Bin, Yao Le, Qian Mingping, He Zhigang, Zou Shaowu, Zhou Bo, Song Zhenshun (2013). Comparison of different methods for the isolation of mesenchymal stem cells from umbilical cord matrix: Proliferation and multilineage differentiation as compared to mesenchymal stem cells from umbilical cord blood and bone marrow. Cell Biology International.

[18] Fong C.Y., Gauthaman K., Cheyyatraivendran S., Lin H.D., Biswas A., Bongso A. (2012). Human umbilical cord Wharton's jelly stem cells and its conditioned medium support hematopoietic stem cell expansion ex vivo. Journal of Cellular Biochemistry.

[19] de Witte SFH, Luk F, Sierra Parraga JM, Gargesha M, Merino A, Korevaar SS (2018). Immunomodulation by therapeutic mesenchymal stromal cells (MSC) is triggered through phagocytosis of MSC by monocytic cells. Stem Cells.

[20] Devaney J, Curley GF, Hayes M, Masterson C, Ansari B, O'Brien T (2013). Inhibition of pulmonary nuclear factor kappa-B decreases the severity of acute Escherichia coli pneumonia but worsens prolonged pneumonia. Crit Care.

[21] O’Croinin Donall F., Nichol Alistair D., Hopkins Natalie, Boylan John, O’Brien Sorca, O’Connor Clare, Laffey John G., McLoughlin Paul (2008). Sustained hypercapnic acidosis during pulmonary infection increases bacterial load and worsens lung injury*. Critical Care Medicine.

[22] Higgins BD, Costello J, Contreras M, Hassett P, O’Toole D, Laffey JG (2009). Differential effects of buffered hypercapnia versus hypercapnic acidosis on shock and lung injury induced by systemic sepsis. Anesthesiology..

[23] Costello J, Higgins B, Contreras M, Chonghaile MN, Hassett P, O'Toole D (2009). Hypercapnic acidosis attenuates shock and lung injury in early and prolonged systemic sepsis. Crit Care Med.

[24] Mareddy Shobha, Broadbent James, Crawford Ross, Xiao Yin (2009). Proteomic profiling of distinct clonal populations of bone marrow mesenchymal stem cells. Journal of Cellular Biochemistry.

[25] Lv Feng-Juan, Tuan Rocky S., Cheung Kenneth M.C., Leung Victor Y.L. (2014). Concise Review: The Surface Markers and Identity of Human Mesenchymal Stem Cells. STEM CELLS.

[26] Danchuk Svitlana, Ylostalo Joni H, Hossain Fokhrul, Sorge Randy, Ramsey Austin, Bonvillain Ryan W, Lasky Joseph A, Bunnell Bruce A, Welsh David A, Prockop Darwin J, Sullivan Deborah E (2011). Human multipotent stromal cells attenuate lipopolysaccharide-induced acute lung injury in mice via secretion of tumor necrosis factor-α-induced protein 6. Stem Cell Research & Therapy.

[27] Gupta Naveen, Krasnodembskaya Anna, Kapetanaki Maria, Mouded Majd, Tan Xinping, Serikov Vladimir, Matthay Michael A (2012). Mesenchymal stem cells enhance survival and bacterial clearance in murineEscherichia colipneumonia. Thorax.

[28] Lee Jae W., Krasnodembskaya Anna, McKenna David H., Song Yuanlin, Abbott Jason, Matthay Michael A. (2013). Therapeutic Effects of Human Mesenchymal Stem Cells inEx VivoHuman Lungs Injured with Live Bacteria. American Journal of Respiratory and Critical Care Medicine.

