# Supplementary figures and images for: Umbilical cord-derived CD362+ mesenchymal stromal cells for E. coli pneumonia: impact of dose regimen, passage, cryopreservation, and antibiotic therapy
Source: Stem Cell Res Ther. 2020 Mar 13;11:116. doi: 10.1186/s13287-020-01624-8 (PMC7071745; doi:10.1186/s13287-020-01624-8)

A

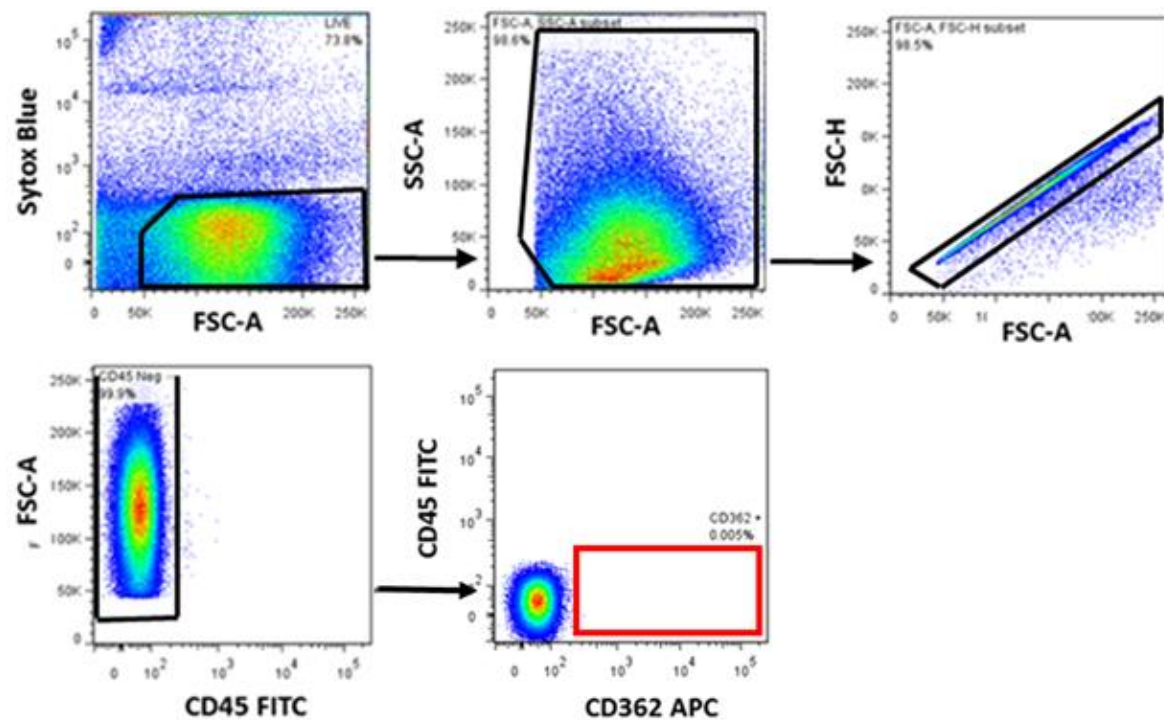

B

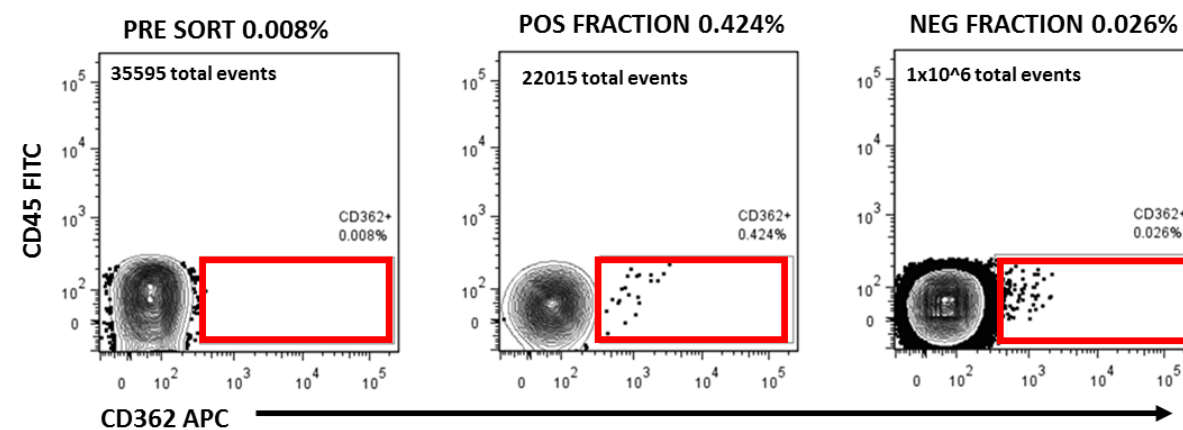

C

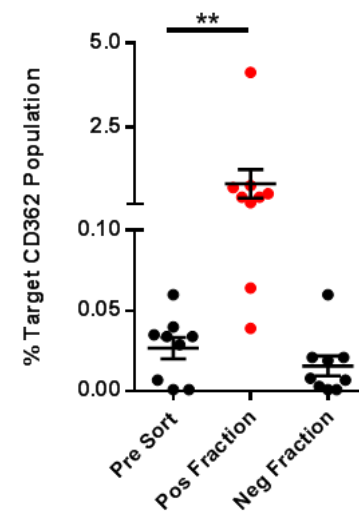

D

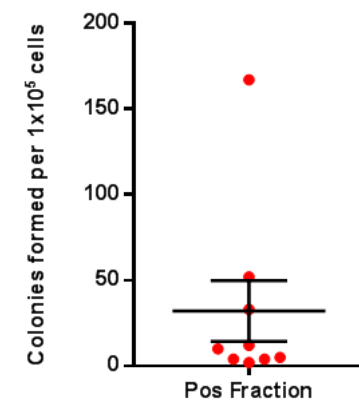

E

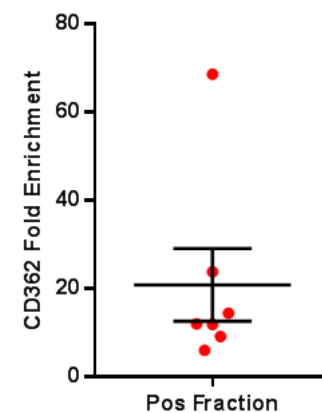

Supplement: Supplementary file 1 — Additional file 1: Supplemental S1. CD362 Isolation from Human Umbilical Cord Tissue. Figure S1. Isolation of CD362+ cells from human umbilical cords tissue by MACs. Figure S2. CD362+ UC-hMSCs reduce the severity of histologic injury following E. coli-induced lung injury. [file 13287_2020_1624_MOESM1_ESM.zip › Figure S1.pdf]

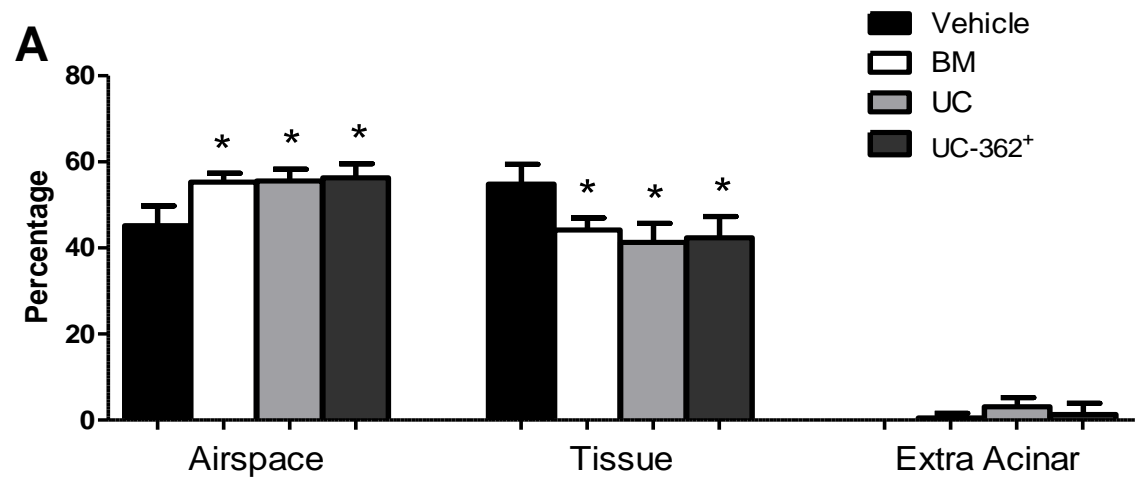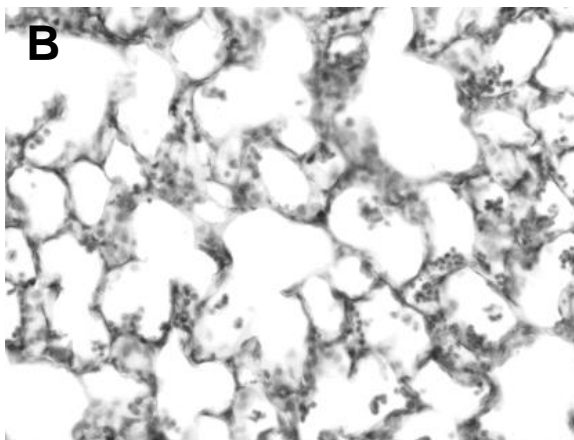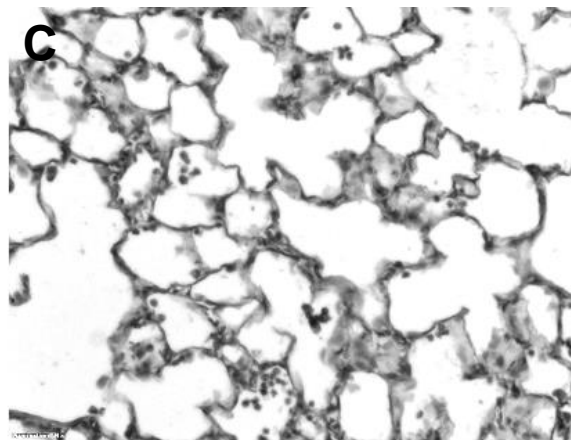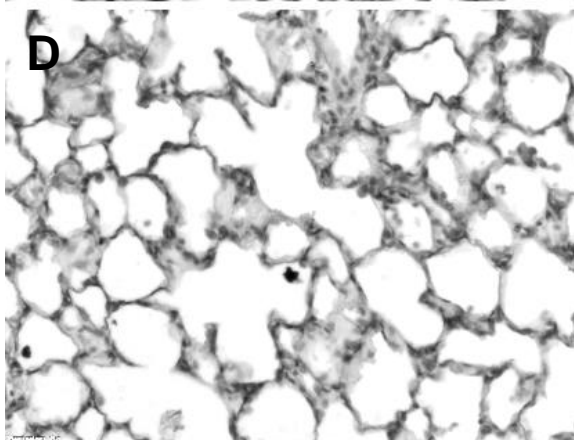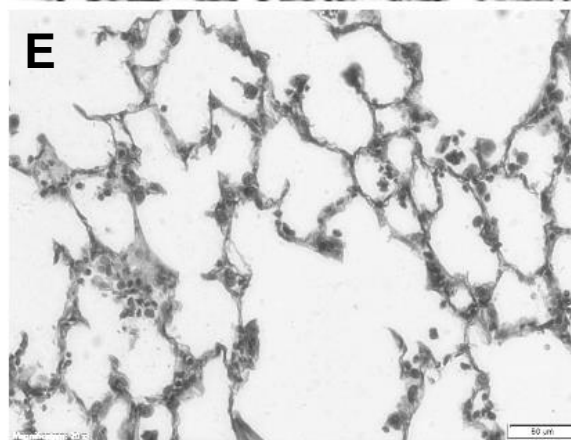

Supplement: Supplementary file 1 — Additional file 1: Supplemental S1. CD362 Isolation from Human Umbilical Cord Tissue. Figure S1. Isolation of CD362+ cells from human umbilical cords tissue by MACs. Figure S2. CD362+ UC-hMSCs reduce the severity of histologic injury following E. coli-induced lung injury. [file 13287_2020_1624_MOESM1_ESM.zip › Figure S2.pdf]
