# Supplementary material for: Umbilical cord-derived CD362+ mesenchymal stromal cells for E. coli pneumonia: impact of dose regimen, passage, cryopreservation, and antibiotic therapy
Source: Stem Cell Res Ther. 2020 Mar 13;11:116. doi: 10.1186/s13287-020-01624-8 (PMC7071745; doi:10.1186/s13287-020-01624-8)
Supplement: Supplementary file 1 — Additional file 1: Supplemental S1. CD362 Isolation from Human Umbilical Cord Tissue. Figure S1. Isolation of CD362+ cells from human umbilical cords tissue by MACs. Figure S2. CD362+ UC-hMSCs reduce the severity of histologic injury following E. coli-induced lung injury. [file 13287_2020_1624_MOESM1_ESM.zip › Supplemental Text.doc]

**Supplemental S1: CD362 Isolation from Human Umbilical Cord Tissue**

Human umbilical cord tissue was collected from virally screened healthy donors by Tissue Solutions Ltd. (Glasgow, UK) or from LifeForce CryoBanks (Altamonte Springs, FL, USA) (all cord tissues provided were obtained according to the legal and ethical requirements of the country of collection, with the approval of an ethics committee and/or similar). In the work reported here, the donor umbilical cord tissue (n=4) had an average weight of 35g and all tissues were transported for processing to Orbsen Therapeutics Ltd. in AQIX solution (Life Science Group Ltd, Barnet, UK) at 4°C. Here, isolations were performed within 48h for UK sourced tissue and max 96h for USA, post birth. Briefly, umbilical cord tissue was washed and the whole tissue was manually dissociated before additional enzymatic digestion in a MEM Alpha (Gibco/ThermoFisher, Loughborough, UK) containing Collagenase 1 (2mg/ml) (Sigma Aldrich, Wicklow, Ireland) at 37°C for controlled amount of time <1h. Once a single cell suspension was obtained by filtration (100µm) cells were stained with CD362-APC (clone 305515; BioTechne, Minneapolis, MN, USA). After 30mins at 4°C the cells were washed and resuspended in MACs buffer 80µl/10^7^cells with the addition of anti-APC beads (Miltenyi Biotec, Bergisch Gladbach, Germany) at a concentration of 20µl/10^7^cells the cells were incubated with beads for 15mins at 4°C. The CD362^+^ cells were then isolated using MS MACs column as per manufactures instructions, (Miltenyi Biotec). Each cell fraction was counted and cells were seeded for expansion. Unselected cell populations, positively selected cells were analysed by flow cytometry (viability dye Sytox, CD45-FITC (clone HI30), BD BioSciences, Oxford, UK), on a BD FACS Canto (BD Biosciences). Data was analysed using FlowJo software (Tree Star Inc., Ashland, OR, USA). Cells populations were expanded to passage 3 and were the analysed for standard ISCT markers of mesenchymal stromal cells on a BD FACS Canto (BD Biosciences) (data not shown).

**Figure S1: Isolation of CD362^+^ cells from human umbilical cords tissue by MACs.** **(A)** Representative gating strategy and analysis used for identification of CD362^+^ stromal cells (viable, CD45^-^) from enzymatically digested whole umbilical cord tissue. CD362^+^ gated population (red box). **(B)** Representative analysis of CD362^+^ population in pre-MACS sort population, positively selected fraction and the remaining negative fraction. **(C)** Target CD362^+^ population in all fractions of umbilical cord cell suspensions; Pre-sort CD362 0.02±0.006, Pos Fraction 0.81±0.42 and Neg Fraction 0.016±0.006 (n=9, % mean±SEM). **(D)** Fold enrichment of CD362^+^ cell population in positive fractions after CD362 MACs selection (20.84±8.23, n=7, mean±SEM). **(E)** Average colonies per 1x10^5^ CD362^+^ cells from positive fraction (32.11±17.76, n=9, mean±SEM).

** Significantly (P<0.01) different from Pre-sort control group.

**Figure S2: CD362^+^** **UC-hMSCs reduce the severity of histologic injury following *E. coli*-induced lung injury.**

CD362^+^ UC-hMSC therapy decreased *E. coli*-induced histologic injury as evidenced by decreased alveolar lung tissue and increased alveolar airspace fractions **(A)**. Representative photomicrographs of lung from a vehicle treated **(B)**, a BM-hMSC treated **(C)**, a UC-hMSC treated **(D)**, and a CD362^+^ UC-hMSC treated animal **(E)**, demonstrate decreased lung injury with heterogenous and CD362^+^ MSCs at 48 hours.

Abbreviations: Vehicle, treatment with vehicle alone; BM, bone marrow derived heterogeneous hMSC; UC, umbilical cord derived heterogeneous hMSC; UC 362^+^, umbilical cord derived CD362^+^ hMSC. Error bars represent standard deviation. n = 5 animals per group. Scale bar is 200μm.

# * Significantly (P<0.05) different from Vehicle control group.
